# An On-Board Shock Absorber Detection Method for General Aviation Aircraft Landing Gears

**DOI:** 10.3390/s26113509

**Published:** 2026-06-02

**Authors:** Chunsheng Li, Haoyu Li, Zongguang Shen

**Affiliations:** Department of Aircraft Engineering, College of Aviation Engineering, Civil Aviation Flight University of China, Chengdu 641419, China; lihaoyu1355@163.com (H.L.); szg18797924608@163.com (Z.S.)

**Keywords:** general aviation aircraft, landing gear, shock absorber detection, rigid-flexible coupling modeling, CNN, LSTM

## Abstract

**Highlights:**

**What are the main findings?**

- Gas leakage primarily reduces initial gas pressure in landing gear shock absorbers, while oleo leakage increases initial gas volume; both affect air spring force.
- A rigid-flexible coupled nose landing gear model incorporating strut flexibility was developed, improving simulation fidelity of dynamic responses during landing.
- A CNN-LSTM deep learning method using only two accelerometers per landing gear achieves over 95% detection accuracy for most fault types under soft and normal landings, and around 90% under heavy landings.

**What are the implications of the main findings?**

- The proposed method offers a practical, sensor-efficient, and maintenance-friendly solution for real-time health monitoring of landing gear shock absorbers.
- It contributes to enhanced operational safety and reduced labor costs for general aviation aircraft.

**Abstract:**

This paper aims to develop an on-board shock absorber detection method for general aviation aircraft. The effects of common gas and oleo leakage are analyzed in this paper. Based on the principle of landing gear dynamics, it is found that gas leakage and oleo leakage would mainly affect air spring force of shock absorbers in various ways. A rigid–flexible coupled landing gear multi-body system (MBS) model is developed by considering strut flexibility, aiming to offer more accurate simulated responses. A database is developed that considers common leakage faults and typical landing conditions using the developed landing gear model. A deep learning model is proposed in this paper. The proposed model is trained and tested using the database simulated from the rigid–flexible coupling landing gear model. The proposed method demonstrates robust detection performance, achieving over 95% precision for most fault types. This work provides a practical, sensor-efficient solution for real-time health monitoring of landing gear shock absorbers, contributing to improved maintenance strategies and operational safety for general aviation aircraft. As this is a preliminary feasibility study, full validation requires future drop tests or instrumented flight tests.

## 1. Introduction

Oleo-pneumatic shock absorbers are widely applied for general aviation aircraft landing gears. Both theoretical analysis and practical experience have demonstrated that the volume of hydraulic fluid and the initial pressure of the gas inside the shock absorber are crucial factors for maintaining the excellent shock absorption performance of the shock absorber [1]. Variations in oleo and gas states would deteriorate landing gear shock absorption performance. Since 2006, a certain flight academy has introduced a large number of Cessna 172R aircraft as main aircraft for the academy’s training flights, and a total of 60 oleo leakage incidents occurred at landing gears of such aircraft between 2007 and 2009, which were mainly caused by seal failure of the shock absorbers [2]. A certain airline has found through statistics that among every 100 aircraft, four aircraft would experience gas leakage in the landing gear shock absorbers. The bottom touch of nose landing gear shock absorber of a certain aircraft occurred frequently during winter seasons due to gas leakage caused by low environment temperature [3,4]. Reference [5] analyzed the main causes of oleo leakage and air leakage in the landing gear of a certain type of aircraft, and it was found that oleo leakage is mainly caused by sealing ring faults, local damage on cylinder, excessive deformation of the cylinder and piston, etc.; gas leakage is mainly caused by weld defects, rubber component aging, oleo pipe defects, incorrect assembly of the screw sleeve, environment temperature, etc. Currently, general aviation aircraft are rarely equipped with sensors mounted inside shock absorbers to monitor oleo leakage and gas leakage, and maintenance engineers would monitor the status of landing gear shock absorbers manually based on the maintenance manual (such as pressure detection, etc.) after the flight mission is completed, which would result in higher labor costs. And given the complex and variable factors contributing to the problem of shock absorber oleo leakage and gas leakage in the landing gear, it is difficult to establish an accurate physical and mathematical model to describe how shock absorbers degrade [1]. This paper aims at developing an on-board oleo and gas leakage detection method for shock absorbers based on landing gear dynamic responses.

Indirect fault detection methods are widely applied for mechanical systems. Based on the relationship between faults and system responses, such methods tend to offer fault detections by analyzing system responses (such as impact signals, acceleration signals, etc.) instead of monitoring factors directly. Such methods are widely applied for bearings [6,7,8], gears [9,10], wheelsets [11,12,13], etc. One main advantage of indirect fault detection methods is that they could offer accurate fault diagnoses based on simple sensor networks. For example, only one accelerometer and one temperature sensor are needed to classify faults occurring on different components of bearing. A direct shock absorber fault detection would require a complex sensor network for the factors affecting shock absorber performance, which need independent sensors for gas, oleo, internal structure, etc. Indirect fault detection methods provide a new perspective to direct various shock absorber faults based on acceleration signals, according to their different influences on landing gear dynamic responses during landing. Nowadays, deep learning methods are widely applied for indirect fault detection.

Deep learning algorithms could adaptively capture the features of raw data through a deep multi-layer network structure, achieving end-to-end fault diagnosis, and are widely applied in the fault diagnosis of mechanical systems [14]. Reference [15] provided a comprehensive review and analysis of the existing fault diagnosis methods for rotary mechanisms based on deep learning, comparing the principles and applications of each deep learning analysis method. Among them, convolutional neural networks (CNNs) have the advantage of extracting features in the spatial dimension and are widely applied in the fault diagnosis of mechanical systems. Reference [16] proposed a rolling bearing fault diagnosis method based on CNN and the skewness index of vibration signals. By converting the vibration indicators of the vibration signals into grayscale images, a CNN was used to extract different fault features of the bearings in the skewness grayscale images, and the accuracy of the diagnosis model reached 99.5%. Reference [17] proposed a fault diagnosis method for planetary gearboxes based on CNNs and vibration bispectrum. This method uses the vibration bispectrum of the original vibration signal as the input of the CNN to enhance nonlinear features and reduce noise. This model has a good recognition accuracy for common gear faults. CNNs are also widely applied in the field of aviation for fault diagnosis. Reference [18] proposed a CNN-based fault diagnosis method for rolling bearings, converting the original vibration signal into an image signal for input into a CNN to achieve fault diagnosis. Reference [19] proposed a one-dimensional CNN fault diagnosis method for aircraft engine gears, extracting fault features from the vibration data using a one-dimensional CNN, and this fault diagnosis method is superior to the support vector machine. Reference [20] proposed a CNN-based fault diagnosis method for aircraft engine bearings, using a one-dimensional adaptive normalization CNN and a one-dimensional physical characteristic-guided CNN combined with the star-crow algorithm to extract the features of multi-dimensional signals of the engine, improving the fault diagnosis effect of aircraft engine bearings. Reference [21] proposed a fault diagnosis method for the main bearing of aircraft engines based on CNNs, obtaining the time-frequency spectrum samples of the original vibration signal through wavelet transform and short-time Fourier transform, and extracting the features of the time–frequency spectrum samples through a CNN with deep and shallow layer feature fusion characteristics to achieve fault diagnosis.

Compared with the advantages of CNNs in extracting features in the spatial dimension, a long short-term memory network (LSTM) can handle the time dependence and long-term memory of signals in the temporal dimension, and can also solve the problems of gradient vanishing and gradient explosion. The combination of CNNs and LSTM can achieve a result of capturing more comprehensive signal features in both the spatial and temporal dimensions. Reference [22] proposed fault diagnosis methods combining CNNs and LSTM, which not only improved the efficiency of feature extraction and reduced the number of parameters, but could also adapt to constantly changing operating conditions. Reference [23] proposed a wind turbine fault detection method based on CNN-LSTM. By using CNNs to extract the dynamic changes in collected data and enhancing LSTM through the attention mechanism, the accuracy of the method was improved. Reference [24] proposed a gearbox fault diagnosis model based on CNN-LSTM, which extracted the fault features of the original vibration signal of the gearbox through the dual channels of CNN and the gated recurrent unit based on LSTM, and classified the fused fault feature vector using Softmax.

Based on the above analyses, in order to ensure the landing gear safety and fully consider the requirements of airworthiness certification, this paper proposes a sensor network which only consists of two accelerometers for each landing gear. By installing acceleration sensors on the fuselage and wheel to collect the dynamic response signals during aircraft landing, and fusing the acceleration signals of the fuselage and wheels into a two-dimensional image signal as the input of the fault diagnosis model, this paper combines the advantages of CNNs and LSTM in feature extraction and proposes a method for monitoring shock absorber oleo leakage and gas leakage of a certain general aviation aircraft landing gear.

## 2. Methodology

The factors causing oleo leakage and gas leakage of shock absorbers in the landing gear are complex and variable, including component production and installation quality, total number of takeoffs and landings, flatness of the runway, ambient temperature, air composition, etc. Oleo leakage and gas leakage are mainly detected via visual inspection with high labor cost. For maintaining a good service quality and operational safety, it is of interest to develop on-board health-monitoring methods which not only enable a rapid leakage detection but also contribute to investigating their influences on shock absorption performance of landing gear and further optimize operation and maintenance strategies.

**Figure 1:**
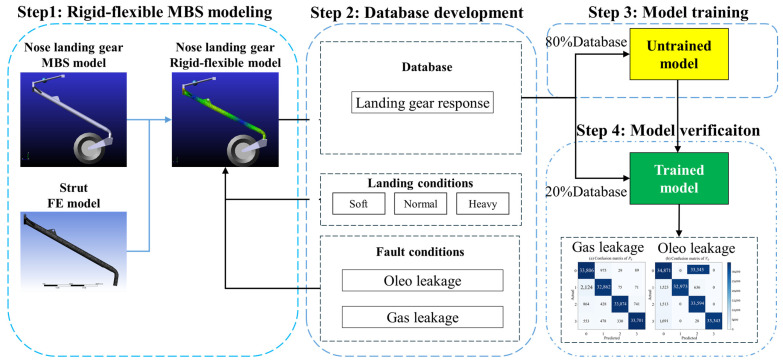
The technique route of this paper.

The performance of a shock absorber is affected by many factors, including gas state, oleo state and interior structure of the shock absorber. A complex sensor network would be required to monitor factors directly; for example, pressure sensor and volume sensor are required to monitor the gas pressure and volume. In addition, some factors are difficult to be monitored. For example, damping the orifice area is crucially important to the damping performance of a shock absorber, and wear and clogging would change the damping orifice area, thereby deteriorating the damping performance of a shock absorber, but it is difficult to monitor the state of damping the orifice area. Based on our research, the key indicators of a shock absorber performance would affect the dynamic responses during landing in various ways, and the dynamic responses are easy to be acquired by sensors and are widely applied in many industries. These findings provide a theoretical basis for the response-based shock absorber fault diagnosis. In addition, most general aviation aircraft are not equipped with landing gear health-monitoring systems, and adding fault detection system for landing gears would have to consider the impact of installing additional sensors on the original structure of the landing gear. Installing additional sensors on shock absorbers directly would destroy the original structure of the shock absorber and reduce the structural strength of the shock absorber. The sensors installed on shock absorbers will work under the environment with high pressure, high temperature and severe vibrations. And the maintenance of this sensor network will require lifting the aircraft fuselage and disassembling the landing gear. This paper proposed a sensor network for shock absorber detection which only needs accelerometers instead of a complex sensor network. As shown in Figure 1, two accelerometers are mounted on fuselage and wheel for each landing gear to acquire vertical acceleration during landing. The wheel accelerometer is installed near the centerline of the wheel axle, while the fuselage accelerometer is mounted on the floor of the cockpit above the shock absorber strut. The fuselage and the wheel acceleration sensors are fixed by adhesive. Each acceleration sensor has a measurement range of ±50 g and a sampling frequency of 1000 Hz. Both accelerometers would work in a more friendly environment, and it would be easier to conduct maintenance for fuselage and wheel accelerometers. Also, in this case, fuselage lift and landing gear disassembling are not required. Such sensor networks will be able to operate with high robustness and lower cost, and will be-maintenance friendly.

Based on the proposed sensor network, a DL model is developed to conduct fault detection of shock absorber leakage due to its advantage of automatic feature extraction, strong nonlinear modeling capabilities and the ability to handle complex, high-dimensional, and time-series data. CNNs and LSTM are applied in the proposed leakage detection method, combining their advantage in acquiring features in space domain and time domain.

A rigid–flexible landing gear model is developed to simulate landing gear dynamic response signals for database to train the proposed DL model. Simulations mainly focus on landing conditions. Oleo leakage and gas leakage would affect the landing gear shock absorption performance. Comparing with takeoff roll, the landing gear shock absorbers would work with a greater working stroke and the landing gears would have more dramatic dynamic responses during landing process. There are more possibilities to detect shock absorber leakage based on dynamic responses during landing conditions than takeoff roll. Hence, landing gear dynamic response, theoretically, could reflect the shock absorber state very well if such a response is well processed. And it would be risky and costly if a shock absorber fault were detected during takeoff roll, because the aircraft would have to terminate takeoff run and reduce speed to 0.0 km/h before the end of the takeoff runway. The passengers on the plane would have to change planes and subsequent departing flights would be affected. So, this paper mainly focuses on simulations of a certain nose landing gear during landing conditions.

As shown in Figure 1, the technique route of this study mainly consists of the following four main steps:

Step 1: For ensuring the accuracy of samples, a rigid–flexible coupled MBS model is developed for the landing gear focused on in this paper to offer samples for database via simulation. Both the shock absorber and the tire are modeled in detail, and the strut is modeled as a flexible body for considering influence of the strut deformation on landing gear dynamic response during landing.

Step 2: Based on the theoretical model of landing gear, the influence of oleo leakage and gas leakage on the shock absorber are discussed and typical fault conditions are decided. Simulations under all typical fault conditions and landing conditions are made to develop dynamic response samples for database.

Step 3: 80% of the developed database is applied for training the proposed DL method.

Step 4: 20% of the untrained samples are fed into the train model to test its performance.

## 3. Rigid–Flexible Coupled Landing Gear Model and Database Development

During the aircraft landing process, the landing gear shock absorption system needs to absorb and dissipate the vertical impact generated by the contact of the wheels with the ground within a short period of time (1 s) after the wheels touch the ground. The factors influencing the vertical impact shock absorption performance of the landing gear mainly include the flight state of the aircraft during landing and the health state of the shock absorber. Among them, the flight state of the aircraft during landing mainly includes the aircraft’s descent speed. Crosswind is one of the main factors affecting the flight state of the aircraft during landing. The direction of the crosswind is mainly lateral and does not directly produce a vertical component. However, the pilot’s correction for the crosswind state (drift method, sideslip method, etc.) would change the lift distribution of the aircraft, resulting in a larger descent rate or a sudden descent at the moment of touchdown. The essence of the impact of crosswind on the shock absorption performance of the landing gear is to change the descent speed of the landing gear at the moment of touchdown. The health state of the shock absorber mainly includes the oil and gas state inside the shock absorber and the internal structural state. As one of the most important methods for analyzing the performance of shock absorbers, landing gear drop dynamics simulation is widely applied in the analysis of landing gear drop performance [25,26,27]. Considering that the shock absorber mainly determines the absorption capacity of the landing gear for vertical impact, landing gear drop dynamics simulation sets the free-fall motion of the landing gear at different initial heights to simulate the dynamic response of the landing gear under different landing states (descent speed, etc.) of the aircraft; and in the landing gear drop simulation, the influence of the material of the landing airport runway on the vertical impact is considered, while the influence of the vibration caused by the smoothness of the runway surface is ignored. This section mainly conducts landing gear dynamics simulation, establishes a landing gear drop dynamics model considering the flexibility of the support rods, compares the dynamics performance of the landing gear before and after considering the flexibility of the support rods, analyzes the influence of structural flexibility on the landing gear, and based on the rigid–flexible coupling model of the landing gear, establishes a landing gear landing dynamic response database.

### 3.1. A Rigid–Flexible Coupled Landing Gear Model

**Figure 2:**
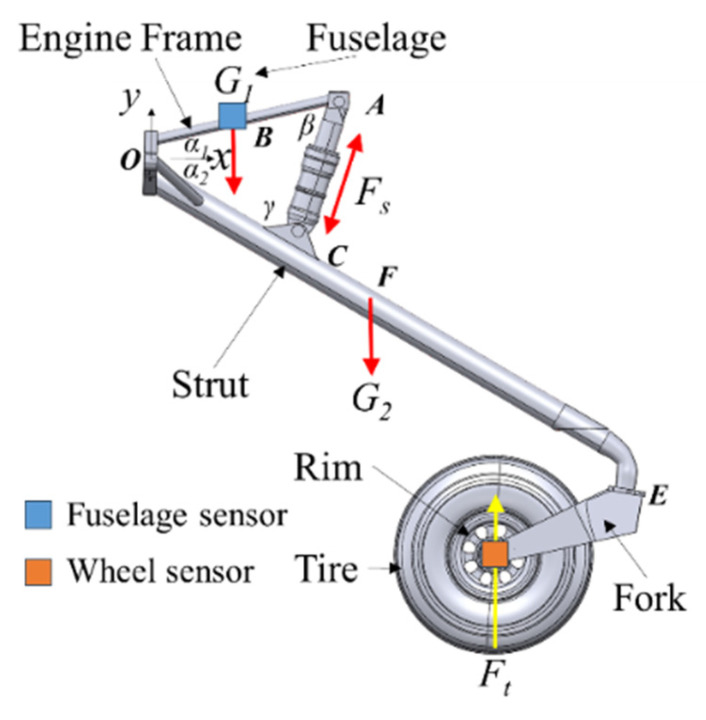
The nose landing gear of a certain general aviation aircraft.

Rigid–flexible coupled dynamic modeling is widely applied in fields such as railways [28,29], robotics [30,31], aerospace [32,33], etc., due to its advantages of being closer to physical reality, high computational efficiency, balance between accuracy and efficiency, stress analysis and fatigue prediction, etc. This paper focuses on a certain transport aircraft, and establishes a rigid–flexible coupled dynamic model of the aircraft’s nose landing gear to simulate the landing gear dynamic responses during the landing process under various conditions. As shown in Figure 2, the nose landing gear considered in this paper mainly consists of the tire, rim, fork, strut, shock absorber, engine frame and fuselage. Such nose landing gear could be modeled as in Equation (1).

(1) $$\left\{\begin{array}{l} M_{1}{\ddot{y}}_{1}L_{OB}\cos\alpha_{1}=M_{1}gL_{OB}\cos\alpha_{1}-F_{s}L_{OA}\cos\beta \\ M_{2}{\ddot{\alpha }}_{2}L_{OF}=M_{2}gL_{OD}\sin\gamma +F_{s}L_{OC}\sin\gamma -F_{t}L_{OE}\cos\alpha_{2} \end{array}\right.$$

where *L_OA_*, *L_OB_*, *L_OC_*, *L_OD_*, *L_OE_* and *L_OF_* are the distance between each force point and the rotation center, o, correspondingly, *L_AC_* is the length of shock absorber in real time, *α*_1_ and *α*_2_ is pitching angle of engine frame and strut, respectively, *β* is the angle between shock absorber and engine frame, *γ* is angle between shock absorber and strut, *F_s_* is the shock absorber axial force, and *F_t_* is the radial force generated by the contact between the wheel tire and the ground.

Equation (2) shows detailed functions of shock absorber axial force *F_s_*(*t*) [31]. As shown in Equation (2), shock absorber axial force *F_s_*(*t*) mainly consists of oleo damping force *F_c_*(*t*), structural limiting force *F_l_*(*t*), air spring force *F_k_*(*t*) and internal friction force *F_f_*(*t*).

(2) $$\left\{\begin{array}{l} F_{s}(t)=F_{k}(t)+F_{f}(t)+F_{c}(t)+F_{l}(t)\\ F_{k}(t)=A_{0}\left[P_{0}{\left(\frac{V_{0}}{V_{0}-A_{0}L(t)}\right)}^{\alpha }-P_{atm}\right]\\ F_{f}(t)=\mu_{p}\frac{4h_{p}}{D}F_{k}(t)\\ F_{c}(t)=\xi \frac{\rho A_{1}^{3}}{2A_{s}^{2}}\cdot \left|V(t)\right|V(t)\\ F_{l}(t)=\left\{\begin{array}{c} k_{l}L(t),L(t)<0\\ 0,0\leq L(t)\leq L_{s\max}\\ k_{l}(L(t)-L_{s\max}),L(t)>L_{s\max} \end{array}\right. \end{array}\right.$$

where *A*_0_ is the effective air pressure area and mainly decided by the inner diameter of the piston rod, *A*_1_ is the oleo pressure area and related to the inner diameter of the shock absorber outer cylinder, *A_S_* is the damping orifice area, *D* is the diameter of the outer cylinder of the shock absorber, *h_p_* is the thickness of the inner cylinder liner of the shock absorber, *k_l_* is the piston stiffness, *L*(*t*) and *V*(*t*) are the real-time stroke and stroke speed of the shock absorber, *L*_max_ is the max stroke of the shock absorber, *P*_0_ is the initial gas pressure, *P_atm_* is the local atmospheric pressure, *V*_0_ is the initial gas volume, *α* is the gas variable coefficient, *ρ* is the oleo density, *ζ* is the fluid damping coefficient of oleo friction loss, and *μ_p_* is the friction coefficient between the inner cylinder liner and the outer cylinder of the shock absorber.

Equation (3) shows the detailed function of radial force generated by the contact between the wheel tire and the ground.

(3) $$F_{t}(t)=(1+C_{T}{\dot{y}}_{t}(t))f(y_{t}(t))$$

where *C_T_* is the vertical damping of tire, $y_{t}(t)$ is the tire compression, and $f(y_{t}(t))$ is the static pressure curve of the tire.

**Figure 3:**
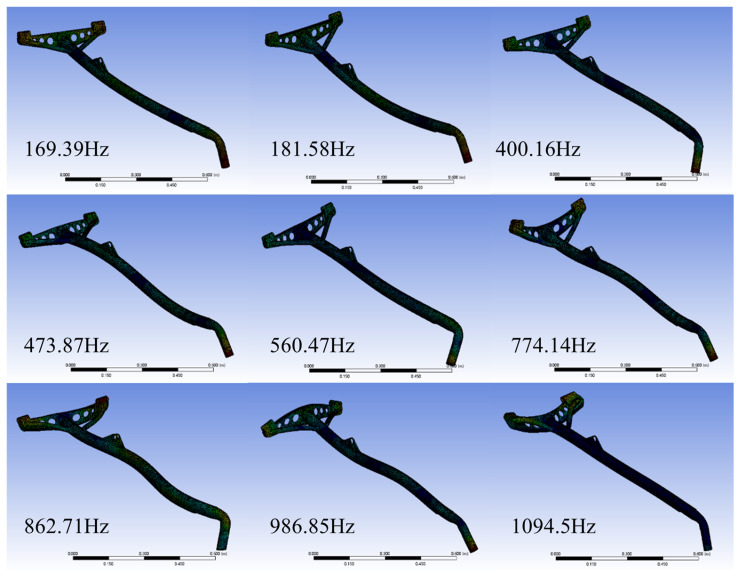
Imported vibration modal of landing gear strut.

The single-engine general aviation aircraft studied in this paper only has the front landing gear equipped with an oil and gas type shock absorber, and the main landing gears are equipped with composite material spring plates. As shown in Figure 2, the nose landing gear is a typical rocking arm structure. The main parameters of the nose landing gear focused on in this paper are shown in Table 1. During the landing process, the strut would rotate at point *o* under the impact force generated by the wheels and the ground, and the strut would inevitably deform when shock absorber works. Accurately describing the flexibility of strut is essential for simulating the dynamic responses of the landing gear during landing conditions. Therefore, to make the simulated results more accurate, modal analyses are carried out on the strut and the results are imported to develop a rigid–flexible coupling dynamic model for the nose landing gear focused on in this paper, as Figure 3 shows. In the simulation of landing gear shock absorption, the airport ground surface adopted the common cement concrete material widely used in general aircraft airports. Considering that the fuel tank of the target aircraft is installed on the wing, and the luggage compartment is set between the wing and the tail wing, all at a certain distance from the front landing gear, the change in fuel quantity before landing and the difference in cargo weight have little impact on the landing shock dynamic performance of the front landing gear.

**Figure 4:**
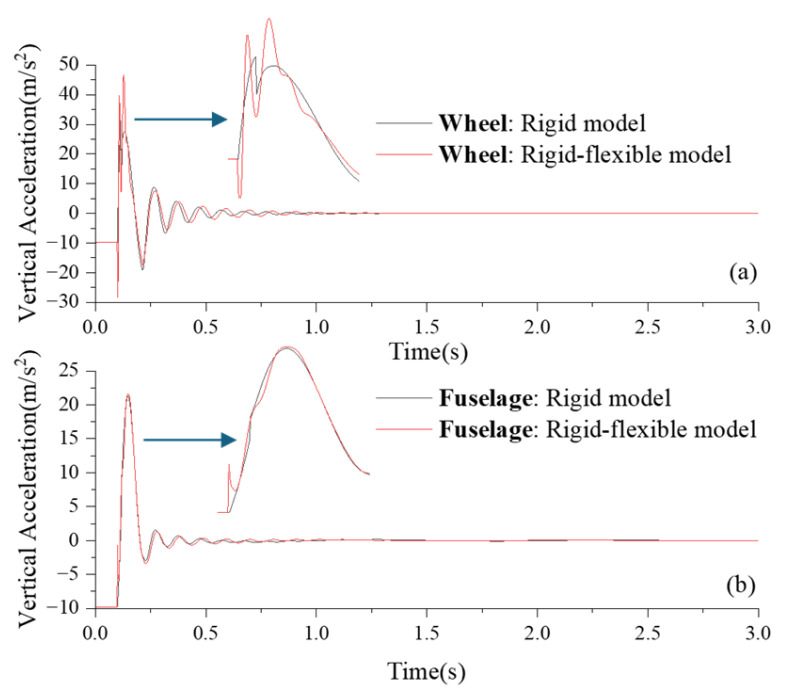
Wheel (**a**) and fuselage (**b**) acceleration from the rigid and rigid–flexible landing gear model.

The current model and method are validated specifically for symmetric landings without crosswind on a rigid, smooth runway. Simulations with and without considering strut flexibility are conducted under a normal aircraft landing condition (descent speed of 1.0 m/s). As shown in Figure 4, wheel and fuselage vertical accelerations are compared for analyzing the influence of strut flexibility on landing gear dynamic responses. The simulation results of the dynamics of the nose landing gear show that within the time range of 0 to 0.121 s, the nose landing gear is in a free-fall stage, maintaining a constant gravitational acceleration value. At 0.121 s, the wheels touch the ground, and the landing gear’s touchdown speed is 1.0 m/s. From 0.121 to 0.126 s, after the wheels touch the ground, the shock absorber and tires are compressed to their maximum stroke, and the wheels experience the maximum peak of vibration acceleration. From 0.126 to 0.214 s, the shock absorber and tires extend, and the wheels experience the maximum reverse peak, with the wheel vibration beginning to oscillate and decay. After 0.6 s, the amplitude of the aircraft body’s vibration acceleration is lower than 0.1 m/s^2^. This meets the relevant requirements in the “Aircraft Landing Gear Strength Design Guide” that “the majority of the impact energy during landing should be absorbed and dissipated within 0.8 s of touchdown, minimizing the impact on the aircraft structure” [34]. And it is found that strut flexibility has more dramatic influence on wheel vertical acceleration than fuselage vertical acceleration. As shown in Figure 4a, after considering strut flexibility, the first peak of wheel acceleration occurred later when the wheel touched the ground, and the amplitudes of the wheel acceleration wave are lower than those without considering strut flexibility. As shown in Figure 4b, considering strut flexibility would reduce the amplitudes of fuselage acceleration subtly.

To test the accuracy of the landing gear model, this paper compares the simulation results with real flight data. Vertical fuselage accelerations from a real flight and landing gear model with and without considering structural flexibility are compared within one second of the aircraft’s wheels touching the ground. As shown in Table 2, the rigid–flexible landing gear model was proved to have higher accuracy than the rigid landing gear model.

Energy absorption and the efficiency factor are two main indicators for evaluating the performance of shock absorbers. Energy absorption refers to the total amount of kinetic energy converted into thermal energy and potential energy by the landing gear shock strut during its compression stroke through oil damping and gas spring deformation. This metric directly quantifies the shock absorber’s capability to dissipate impact energy, typically expressed in joules (J). The efficiency factor is defined as the ratio of the actual energy absorbed by the shock strut to the ideal energy that would be absorbed under a constant load throughout the compression stroke. Alternatively, it can be expressed as the ratio of the mean load to the peak load during compression. This metric indicates the ability of the shock absorber to transmit impact loads smoothly into the airframe. A higher efficiency (typically between 70% and 90%) results in a lower peak load for the same absorbed energy, thereby reducing fatigue damage to the landing gear and fuselage and improving ride quality. Equations (4) and (5) show the functions of energy absorption (*W*) and efficiency factor (*η*), respectively.

(4) $$W=\sum P_{z}\cos\alpha \cos\beta \Delta S$$

(5) $$\eta =\frac{W}{{\left(P_{z}\cos\alpha \cos\beta \right)}_{\max}S_{\max}}$$

where *α* and *β* are the pitching angle and rolling angle of fuselage, *P_z_* is the vertical shock absorber load, ΔS is the variation in the travel distance of the shock absorber during this period and $S_{\max}$ is the max stroke of the shock absorber.

**Figure 5:**
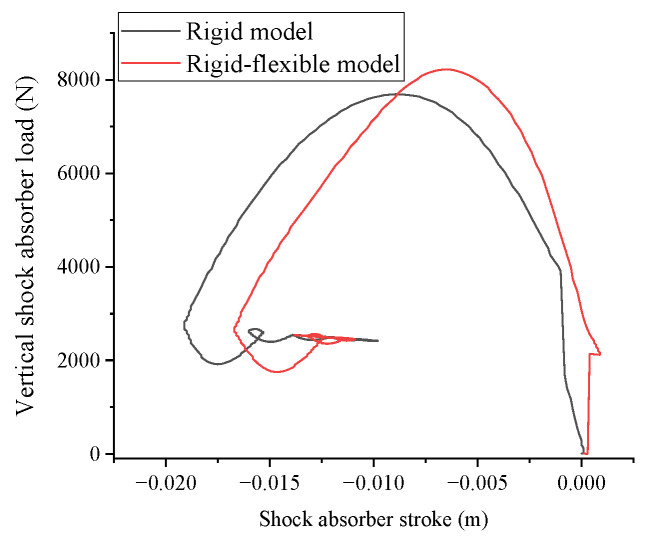
Shock absorber stroke–load curves from the rigid and rigid–flexible landing gear model.

Figure 5 shows the stroke–load curve with and without considering strut flexibility under the vertical decent speed of 1.0 m/s. The energy absorption and efficiency factor of the shock absorber are derived based on Figure 5. As shown Table 3, the strut flexibility has a subtle influence on the energy absorption and efficiency factor of the shock absorber. As shown in Figure 5, the strut flexibility would reduce the max stroke and increase the max vertical load of the shock absorber simultaneously.

**Figure 6:**
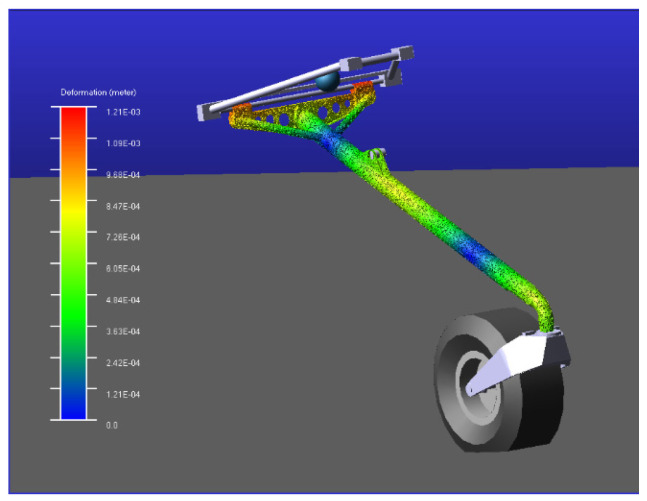
Strain distribution of strut when wheel touching down.

Figure 6 shows the strain distribution of strut before and after considering the strut flexibility, and it is found that the max stress area occurs at the junction of the strut and the engine frame. It is proved that the flexibility of strut would affect the dynamic response of landing gear during landing by absorbing certain vibrations, and the reduced wheel and fuselage acceleration would cause more challenges to the shock absorber health-monitoring method.

### 3.2. Typical Shock Absorber Fault Conditions

Based on the theoretical model of the shock absorber (Equation (2)), the axial force of the shock absorber is mainly determined by the internal structure and the state of sealed oleo and gas. As shown in Equation (2), oleo damping force *F_c_*(*t*) is mainly decided by the sectional area inside the shock absorber, which would not have dramatic variations during real operation, density and damping co-efficiency of oleo, which are usually considered as constant and would not vary with oleo or gas leakage. The structural limiting force *F_l_*(*t*) is mainly determined by the material of the shock absorber piston and outer cylinder, which would not vary with oleo or gas leakage. Air spring force *F_k_*(*t*) and internal friction force *F_f_*(*t*) are mainly determined by initial gas volume and initial pressure. Considering that the shock absorbers are filled with oleo and gas inside, the initial gas volume would be affected (increased) by oleo leakage, and the initial gas pressure would be affected (reduced) by gas leakage. Hence, it is found that oleo leakage and gas leakage would have limited influence on oleo damping force *F_c_*(*t*) and structural limiting force *F_l_*(*t*), but would affect air spring force *F_k_*(*t*) and internal friction force *F_f_*(*t*) via changing the initial gas volume and initial pressure.

**Figure 7:**
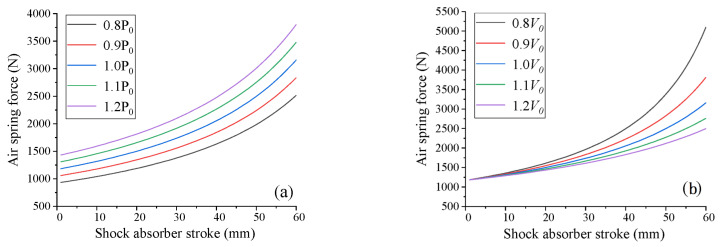
Fault conditions considered in this paper: (**a**) initial gas pressure (*P*_0_) faults and (**b**) initial gas volume (*V*_0_).

In this paper, faults of initial gas volume and initial gas pressure are focused on in this paper to develop health-monitoring methods for oleo leakage and gas leakage of shock absorbers. Four different fault levels are considered for initial gas volume fault and initial gas pressure fault. As analyzed above, such faults would affect air spring force *F_k_*(*t*) and internal friction force *F_f_*(*t*) in a different way. As shown in Figure 7, air spring force *F_k_*(*t*) would increase with the increase in initial gas pressure, and reduce with the increase in initial gas volume [35]. The initial gas pressure faults would cause linear variation in the air spring force of the shock absorber. The initial gas volume faults would cause nonlinear variation in the air spring force of the shock absorber; for the same initial gas volume fault, air spring force would show larger variation under a larger shock absorber stroke.

### 3.3. Database Development

Based on the rigid–flexible landing gear model, a database is developed to train and test the model proposed in this paper. Both initial gas pressure faults and initial gas volume faults are selected to represent oleo leakage and gas leakage. It is assumed in this paper that the volume inside a shock absorber would remain constant; so, oleo leakage would affect gas volume, thereby affecting the volume of gas, and gas leakage would affect the gas pressure. According to the manual, for most general aviation aircraft, when the initial gas pressure fluctuation range of the oleo-pneumatic shock absorber reaches ±5%, the status of the shock absorber needs to be monitored; when the initial gas pressure fluctuation range reaches ±10%, the oleo-pneumatic shock absorber must be refilled with gas; when the initial gas pressure fluctuation range reaches ±20%, the aircraft cannot be released and the shock absorber needs to be refilled with gas and undergo a sealing inspection [36]. Hence, four levels are considered for both initial gas pressure faults and initial gas volume faults, including ±10% and ±20% parameter fluctuation representing mild fluctuation and severe fault conditions correspondingly. According to the ideal gas law, the effect of ambient temperature on oleo-pneumatic strut performance is essentially manifested through changes in initial gas pressure (*P*_0_). For general aviation aircraft operating within a typical temperature range (−20 °C to +45 °C), the corresponding variation in *P*_0_ is approximately ±8% (based on the gas state equation). This range is fully covered by the predefined fault levels.

Besides shock absorber states, the state of the aircraft before touching the ground also affects the landing dynamics response of the landing gear. According to relevant regulations, for small single-engine general aviation aircraft, the ideal landing descent speed is 0.5 m/s to 1.0 m/s; a landing descent speed of 1.0 m/s to 1.5 m/s would be considered a heavy landing, which would be judged as having a tendency for medium landing in flight training; when the landing descent speed reaches 1.524 m/s, the maintenance engineers need to inspect the landing gear and the fuselage skin; when the landing descent speed is 1.524 m/s, it is a serious heavy landing and a comprehensive flaw detection inspection must be carried out [37]. Based on this, three typical aircraft-landing conditions are considered in this paper, including descent speeds of 0.5 m/s, 1.0 m/s and 1.5 m/s, representing a soft landing, a normal landing and a heavy landing.

Both fault conditions and landing conditions are put into the rigid–flexible landing gear model for simulating dynamic responses under all considered conditions. Fuselage vertical acceleration signal and wheel vertical acceleration signal are obtained via simulations for building database. Simulations are made for acquiring dynamic responses of the first 6.0 s during landing progress and the sampling frequency of virtual sensors is set as 1000 Hz.

## 4. Model Training and Verification

### 4.1. Technical Route

This paper proposed an on-board health-monitoring method for landing gear leakage detection by combining the advantages of CNN and LSTM. A network structure of “two-layer CNN + two-layer LSTM” is adopted in the deep learning method proposed in this paper. The first layer of CNN is mainly applied for local feature extraction of the original acceleration in time series, capturing the short-term local fluctuation features of the acceleration signal (such as the instantaneous peak of landing impact, local waveform distortion caused by abnormal damping). The second layer of the CNN further abstracts and integrates the local features extracted by the first layer, extracting higher-level global local features (such as the feature waveforms corresponding to different fault modes, the associated features of multiple shock signals). This enhances the robustness of the features, filtering out the noise in the original signal (such as environmental vibrations during landing and sensor noise). Using a two-layer CNN can accurately capture the instantaneous impact, waveform distortion, etc., corresponding to the shock absorber fault, and significantly reduces the model parameters, avoiding overfitting, and adapting to small-sample fault diagnosis scenarios (aircraft fault data collection entails high costs and the sample size is small). The first-layer LSTM receives the local temporal features extracted by CNN to model the dependency relationship between adjacent time steps, learn the temporal evolution pattern of the acceleration signal (such as the dynamic changes in the buffer from impact to stability during landing, and the temporal abnormal trend caused by faults), and retain the temporal dimension, providing the second-layer LSTM with features with temporal context. The second-layer LSTM performs full-long-term dependency modeling on the temporal features of the first layer, integrating the temporal information of the entire landing process, and finally outputs a global temporal feature vector (compressing the temporal dimension and retaining the global features), capturing the long-term cumulative effect of faults (such as the acceleration waveform shift in the entire landing process caused by faults, and multi-stage abnormalities), which is a global temporal feature that CNN cannot efficiently model. In the designed “two-layer CNN + two-layer LSTM” structure, the CNN is mainly responsible for extracting short-term local features (impacts, waveform distortions), while the LSTM is mainly responsible for modeling long-term sequence global dependencies (time evolution, cumulative anomalies). CNNs and LSTM complement each other and comprehensively cover the feature dimensions of shock absorber faults. The manifestations of shock absorber faults include instantaneous impact anomalies (the advantage of CNN) and long-term waveform deviations (the advantage of LSTM). This structure perfectly matches the characteristics of fault features. For the case where the input is the dual acceleration signals of the wheels and the fuselage, the CNN + LSTM structure can effectively integrate the features of the two sensors and improve the diagnostic accuracy. The structure of “two-layer CNN + two-layer LSTM” has fewer parameters and faster training speed, and is suitable for deployment in airborne diagnostic systems (with high real-time requirements) while ensuring diagnostic accuracy.

**Figure 8:**
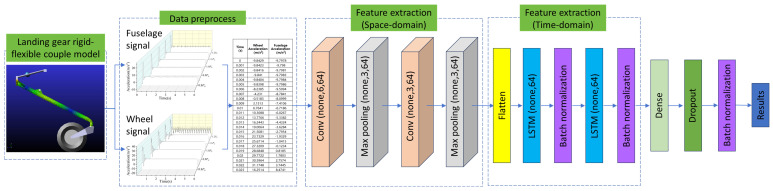
The architecture of the proposed health−monitoring method.

As shown in Figure 8, raw fuselage and wheel acceleration signals are aligned, structured, and normalized into a tensor input of shape (none, 3, 1000) for the network. The spatial-domain feature extraction module employs two stacked 1D convolutional layers (Conv) paired with max-pooling layers: the convolutional layers extract local, short-term spatial features (e.g., transient impact peaks, waveform distortions caused by shock absorber faults) via local receptive fields and weight sharing, while the max-pooling layers downsample the temporal dimension from six to three, reducing computational complexity and enhancing feature robustness against noise. Following this, the time-domain feature extraction module processes the CNN-derived features with a Flatten layer to reshape the tensor, followed by two stacked LSTM layers interspersed with Batch Normalization (BN) layers: the LSTM layers model long-term temporal dependencies and global temporal evolution of the acceleration signals (e.g., cumulative fault-induced waveform deviations across the entire landing process), while the BN layers accelerate network convergence, mitigate gradient vanishing/exploding, and improve generalization. Finally, the classification head consists of a Dense (fully connected) layer to map high-level features to the fault classification space, a Dropout layer to prevent overfitting (critical for small-sample fault diagnosis scenarios), an additional BN layer for feature stabilization, and an output layer that produces the final fault diagnosis results. This hybrid CNN-LSTM architecture synergistically combines the strengths of CNNs in local spatial feature extraction and LSTMs in long-sequence temporal modeling, enabling comprehensive capture of both transient and cumulative fault characteristics, while regularization techniques (Dropout, BN) ensure robust performance and generalization in complex aircraft landing vibration environments.

As shown in Table 4, the epoch for model training is set as 200. Learning rate decay is applied to prevent a situation where, in the later stage of model training, the value of the loss function keeps fluctuating around the minimum and is difficult to reach the optimal level. After every five epochs, the learning rate decay is reduced to 0.01 of its original value. ReLU is selected due to its advantage for classification. To alleviate the problem of model overfitting, Dropout is applied in the final fully connected layer of the classification task, and the Dropping rate is set as 0.5.

This paper tests and compares the performance of the proposed model after considering the strut flexibility of landing gears via the following steps:

(1) The sensor-acquired signals from the rigid–flexibility MBS (multi-body system) landing gear model are merged as a 3 × 1000 data matrix consisting of time, wheel and fuselage vertical acceleration. Such merged matrix is fed into the proposed model to enable the model to learn the underlying patterns and dynamics associated with different fault conditions.
(2) Then, 80% of the simulated database developed in Section 3 is used to train the proposed model. Such a training process tends to enable the model to learn the underlying patterns and dynamics associated with different fault conditions.
(3) The remaining 20% of the untrained database is used to test the performance of the trained model by acquiring accuracy and precision from the confusion matrix. Such a testing phase tends to explore the performance of the proposed model for new or unseen time-series data.
(4) In order to investigate the superiority of the proposed model, the test results of the proposed model trained by the database simulated based on the rigid–flexible landing gear model are compared with results of the proposed model, CNNs and LSTM trained by the database simulated based on the rigid landing gear model.

### 4.2. Results

**Figure 9:**
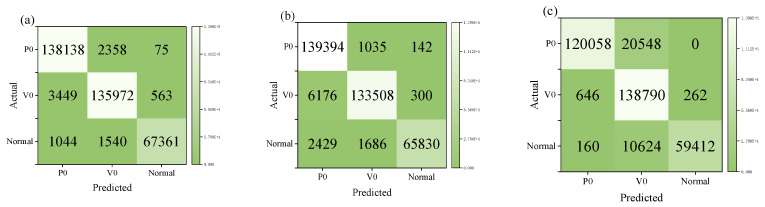
Confusion matrix of the proposed method under the landing speed of 0.5 m/s (**a**), 1.0 m/s (**b**) and 1.5 m/s (**c**).

After following the technique route introduced above, the proposed model is trained and tested in Python 3.10 on a computing device with a 12th Gen Intel Core i5-12400KF CPU and an NVIDIA GeForce RTX 4060 GPU graphic processing unit. After training and testing, the confusion matrices of the proposed model are derived under all considered conditions, as shown in Figure 9. In the derived confusion matrix, these labels are considered, including initial gas pressure (*P*_0_) faults, initial gas volume (*V*_0_) faults and normal condition (Normal).

Based on the confusion matrices shown in Figure 9, two indices are considered in this paper to estimate the performance of the proposed model trained by the database simulated by the rigid–flexible landing gear model, which are *precision* and *accuracy*. As shown in Equation (6), *precision* refers to the proportion of samples that are actually positive among all samples predicted as positive, and it measures the reliability of the model’s prediction of positive classes; *accuracy* is applied to evaluate the ability to judge the whole sample correctly by calculating the proportion of correctly classified samples in the total number of samples.

(6) $$\left\{\begin{array}{l} Precision=\frac{TP}{TP+FP}\times 100\%\\ Accuracy=\frac{TP+FN}{TP+TN+FP+FN}\times 100\% \end{array}\right.$$

**Figure 10:**
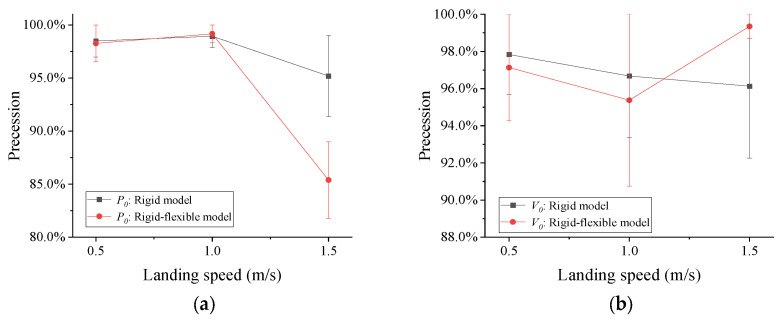
Precision of initial gas pressure fault detection (**a**) and initial gas volume fault detection (**b**).

Figure 10 shows the *precision* of the proposed model for initial gas pressure faults and initial gas volume faults, with and without considering strut flexibility. As shown in Figure 10, strut flexibility would have a different influence on precision for initial gas pressure faults and initial gas volume faults. As shown in Figure 10a, for soft (0.5 m/s) and normal (1.0 m/s) landing conditions, the *precision* of the proposed model for initial gas pressure faults with and without considering strut flexibility are nearly the same and all above 98.0%. For the heavy-landing condition (1.5 m/s), strut flexibility would reduce the *precision* of the proposed model for initial gas pressure faults from 95.2% to 85.3%. The initial gas pressure *P*_0_ of the shock absorber is a global and static state parameter. Its failure nature is the deviation of the pressure reference value in the air chamber, and its impact on the acceleration signal is a global, gradual trend change (such as a small offset of the overall impact peak, and the overall change in the buffering force), rather than a local, instantaneous waveform distortion. The rigid body model of the landing gear only simulates the rigid body motion of the landing gear, and the acceleration characteristics brought by the change in *P*_0_ are a single and clear rigid body dynamic response: the differences in the acceleration waveforms corresponding to different *P*_0_ are clear, the feature boundaries are distinct, and the deep learning model can easily distinguish them. Therefore, even at high speeds, it can maintain an accuracy of over 95%. The rigid–flexible coupling model introduces elastic deformations of structures such as support rods, which would generate a large number of additional flexible vibration modes, structural resonances, and nonlinear elastic forces. These flexible features will be superimposed on the global trend changes brought by *P*_0_, equivalent to “adding noise” to the clear fault features, blurring the feature boundaries between different *P*_0_ values. A descent speed of 1.5 m/s is close to a heavy landing, which is a high-impact condition. The elastic deformation and vibration response of the landing gear structure will be significantly amplified in this case, and the nonlinear and time-varying characteristics of the flexible features are fully highlighted: at this time, the vibration energy brought by the structural flexibility is much greater than the dynamic response change brought by the *P*_0_ fault feature, and the *P*_0_ fault feature is completely “buried” in the flexible vibration, making it difficult for the deep learning model to extract the effective features corresponding to *P*_0_ from the complex mixed signals, resulting in a diagnostic accuracy of about 85% at this time; while the rigid body model is free from the influence of flexibility, the feature of *P*_0_ remains clear, only slightly attenuated due to the nonlinearity of the high-speed impact; so, the accuracy is much higher than that of the rigid–flexible coupling model.

As shown in Figure 10b, for soft (0.5 m/s) and normal (1.0 m/s) landing conditions, the *precision* of the proposed model for initial gas volume faults with and without considering strut flexibility is all above 95.0%, though the strut flexibility would reduce the *precision* of the proposed model for initial gas volume faults; for the heavy-landing condition (1.5 m/s), the strut flexibility would increase the *precision* of the proposed model for initial gas volume faults from 96.1% to 99.3%. The initial gas volume *V*_0_ of the shock absorber directly determines the stiffness, damping, and buffer stroke of the shock absorber, which are the core structural characteristics of the shock absorber and are parameters strongly coupled with the dynamic response of the structure. The essence of its failure is the structural change in the shock absorption characteristics of the shock absorber, and its impact on the acceleration signal is local, and causes instantaneous distortion of the shock waveform and changes in the structural vibration mode, rather than a global trend. In a high-impact condition with a 1.5 m/s descent speed, the flexible characteristics are amplified under strong impact, and the *V*_0_ feature is easier to distinguish: The change in *V*_0_ directly affects the buffer stroke and stiffness of the shock absorber. The differences in structural elastic deformation, resonance frequency, and vibration energy corresponding to different *V*_0_ values will be amplified by the high-speed impact, forming highly distinguishable features. The deep learning model can accurately extract these local and instantaneous flexible vibration features; so, the diagnostic accuracy in high-speed conditions surges to nearly 100%, far higher than that of the rigid body model without flexible features.

**Figure 11:**
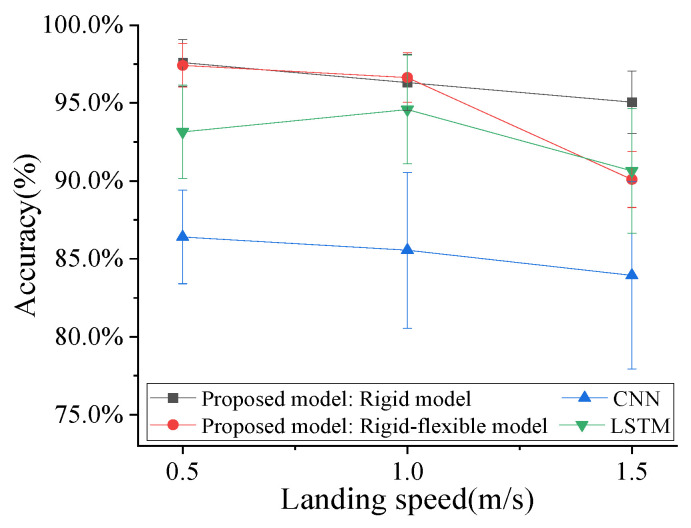
*Accuracy* comparisons between the proposed model and common methods.

The *accuracy* of the proposed model trained by the database simulated from the rigid–flexible landing gear model is derived. The *accuracy* of the proposed model with and without considering strut flexibility is compared with CNNs and LSTM. Both CNNs and LSTM are properly tuned during model training and model testing based on the database developed in this paper. As shown in Figure 11, the *accuracy* of the proposed model under all three landing conditions is above 90.0% after considering strut flexibility. For soft- and normal-anding conditions, the *accuracy* of the proposed model maintains good performance for two fault types after considering strut flexibility, which is above 96.0% and better than that of CNNs and LSTM. For the heavy-landing condition, the *accuracy* of the proposed model would reduce to 90.0% from 95.0% after considering strut flexibility, which is close to that of the CNN but better than that of LSTM. According to relevant regulations for small single-engine general aviation aircraft, when the landing descent speed reaches 1.524 m/s, the maintenance engineers need to inspect the landing gear and the fuselage skin. When the descent speed of the target aircraft approaches 1.5 m/s, pilots and maintenance crew should first check if there is a *V*_0_ fault alarm. If no *V*_0_ fault alarm occurs, the aircraft maintenance personnel should inspect the air pressure status of the shock absorber and conduct damage detection on the landing gear structure to ensure the health state of the landing gear.

## 5. Discussion

**Figure 12:**
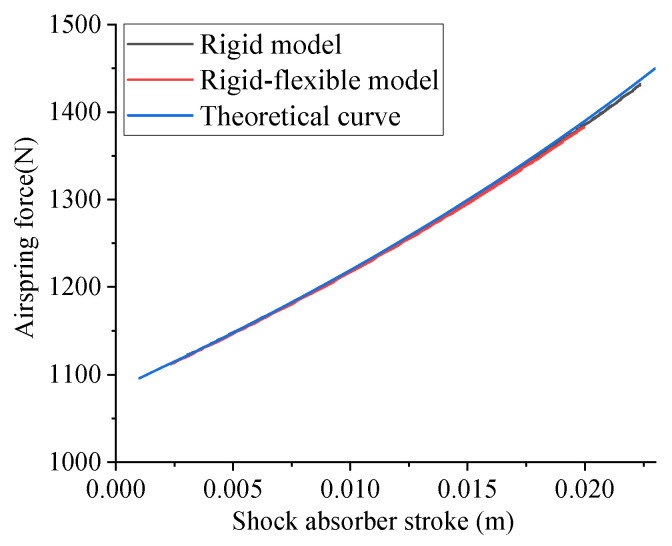
Comparisons of shock absorber air spring force.

This paper develops a dynamic model of the landing gear shock absorption system of a certain transport aircraft to preliminarily verify the feasibility of the diagnosis method for the leakage of the landing gear oleo and gas shock absorber. Considering that the shock absorber mainly affects the vertical impact absorption and dissipation of the landing gear, the dynamic model of the landing gear established in this paper mainly focuses on the vertical vibration response. To ensure the accuracy of the landing gear dynamics simulation, the shock absorber dynamics modeling adopted the principal model that is widely used in the landing gear drop dynamics modeling. As shown in Figure 12, the simulation results of the landing gear dynamics air spring with and without considering the flexibility of the strut are consistent with the principal model, indicating that the landing gear dynamics model can accurately reflect the working principle of the shock absorber.

The current model has limitations in simulating complex working conditions such as symmetrical landing and asymmetric loads during the actual landing process of the aircraft. To verify the generalization of the proposed diagnosis method for the leakage of the landing gear oil and gas shock absorber, future research work will optimize the landing gear model and expand the sample library by increasing the simulation working conditions dimensions, including developing a complete model of the aircraft including all three landing gears, considering the flexibility of wings and fuselage, simulating the landing process of an aircraft with three landing gears, etc.

This paper initially verified the accuracy of the landing gear model by comparing the simulation data with the existing onboard recording data of the aircraft. Although the comparison results were relatively close, due to the limited onboard data information of the target aircraft (only the aircraft body acceleration, and the signal sampling frequency is low), there are limitations in the model verification. To better verify the accuracy of the model, the next stage of research will verify the accuracy of the landing gear model through the flight experiments of the target aircraft. Considering that the target aircraft does not have landing gear sensors, a sensor network was designed to meet the aircraft flight experiment requirements. This sensor network is specifically designed for the detection of the front landing gear, and one single-axis acceleration sensor needs to be installed on each of the left and right sides of the fuselage floor and the wheel axle of the front landing gear, totaling two sensors. The overall design must comply with the aeronautical regulations of CCAR-23 [38] and the environmental adaptability and reliability requirements of RTCA DO-160G [39]. Sensor selection must meet the core specifications of ±50 g measurement range, 1000 Hz sampling frequency, 0–2000 Hz frequency response, etc., and meet the DO-160G certification requirements of MEMS capacitive accelerometers. It needs to pass a complete set of environmental tests including temperature, vibration, shock, EMC, and salt spray, and meet the requirements of anti-vibration, anti-shock, and temperature drift compensation, with a zero bias temperature coefficient of ≤0.5 mg/°C and a zero bias variation in the full temperature range of ≤±15 mg. Installation uses double-sided tape + epoxy structural adhesive reinforcement method. The sensor on the fuselage floor is installed above the shock absorber of the front landing gear at the reinforced area, and the wheel axle sensor is additionally equipped with waterproof sealing protection. The sensitive axis needs to be precisely aligned with the aircraft Z-axis, and the installation angle error is ≤±2°. The power supply uses the aircraft’s 28 V DC bus, with a voltage range of 18–32 V DC, and is equipped with reverse connection, overvoltage, surge, etc., protection circuits. Data transmission uses analog signals + distributed data acquisition unit + ARINC 429 bus architecture. The data acquisition module needs to meet ≥4 channels, 1000 Hz/channel sampling rate, and ≥16-bit ADC resolution requirements. Cables and connectors use aviation-grade standards. The entire network architecture is simple, maintenance-friendly, follows CCAR-23, DO-160G and other standards, has a clear aeronautical path, and can meet the detection requirements of general aviation aircraft.

## 6. Conclusions

Oleo leakage and gas leakage would threaten the performance of landing gears, and have a negative influence on passenger comfort and aircraft safety. This paper aims at exploring an on-board detection method for oleo leakage and gas leakage by analyzing how oleo leakage and gas leakage would affect the landing gear system and considering the flexibility of the main component for simulation. As a preliminary feasibility study, this work demonstrates the methodological potential under simplified conditions. The work in this paper is concluded as follows:

(1) This paper first examines common oleo leakage and gas leakage conditions and analyzes how oleo and gas leakage would affect the performance of landing gear shock absorption system. Based on the principle of landing gear dynamics, oleo leakage and gas leakage mainly affect air spring force by varying initial gas pressure *P*_0_ and initial gas volume *V*_0_ of shock absorbers. This paper presents an on-board health-monitoring method for oleo leakage and gas leakage by analyzing landing gear dynamic responses during landing.
(2) In this paper, a rigid–flexible coupled landing gear model considering strut flexibility is developed to simulate the landing gear dynamic responses during landing and to build the training database for the proposed method with samples closer to real conditions.
(3) The proposed method is trained by the simulated database considering strut flexibility. Both *accuracy* and *precision* are selected to evaluate the performance of the proposed method after considering strut flexibility. Based on analyses, the strut flexibility would have a different influence on the *precision* of the proposed method for initial gas pressure faults and initial gas volume faults under heavy-landing conditions. The *accuracy* of the proposed method remains high-level after considering strut flexibility for soft- and normal-anding conditions, but would reduce to 90.0% under heavy-landing conditions.

Future studies will focus on optimizing landing gear models, which would simulate the dynamic response during whole landing conditions and would consider more operational variabilities, such as runway conditions, environmental conditions during landing, aircraft mass conditions, etc. The performance of the proposed detection method will be improved, especially for heavy-landing conditions. Field tests will be conducted to enrich the database from practical flights, including on-board monitoring system design and corresponding airworthiness certification documents, signal denoise and analysis method, flight test plan design, etc. Both field test data and historical maintenance records will be applied to train the optimized model and conduct transfer learning and model fine-tuning to evaluate the performance degradation of the model from simulation to practical application. Complex coupling fault conditions will be considered, including the simultaneous deterioration of stiffness and damping due to structural wear, changes in damping characteristics caused by oil contamination and time-varying internal friction coefficients, etc.

## Figures and Tables

**Table 1:** Landing gear main parameters.

Symbol	Value	Unit
*A*_0_	638.0	mm^2^
*A*_1_	1885.0	mm^2^
*A_S_*	8.0	mm^2^
*C_T_*	1000.0	N·s/m
*D*	9.7	mm
*k_m_*	32.0	mm
*L*_smax_	60.0	mm
*P*_0_	1,930,531.96	pa
*P_atm_*	95,100.0	pa
*V*_0_	65,000	mm^3^
*a*	1.1	/
*r*	873.0	kg/m^3^
*z*	2.0	/

**Table 2:** Comparisons of real flight data and simulation data.

Descent Speed	Real Flight	Rigid Model		Rigid–Flexible Model	
		Value	Error	Value	Error
0.5 m/s	2.73 m/s^2^	2.45 m/s^2^	10.3%	2.62 m/s^2^	4.0%
1.0 m/s	4.48 m/s^2^	4.41 m/s^2^	1.56%	4.43 m/s^2^	1.11%

**Table 3:** Energy absorption and efficiency factor with and without considering strut flexibility.

Energy Absorption (J)		Efficiency Factor	
Rigid Model	Rigid–Flexible Model	Rigid Model	Rigid–Flexible Model
97,090	97,640	0.6539	0.6746

**Table 4:** Main parameters for model training.

Parameter	Value
Epoch	200
Initial learning rate	0.0001
Learning rate decay	0.01
Batch size	256
Activation Function	ReLu
Dropout rate	0.5

## Data Availability

Data will be made available on request.

## Abbreviations

The following abbreviations are used in this manuscript:

CNNs	Convolutional neural networks
DL	Deep learning
LSTM	Long short-term memory network
SDP	Symmetrized dot pattern
FN	False negative
FP	False positive
TN	True negative
TP	True positive
